# Does unequal economic development contribute to the inequitable distribution of healthcare resources? Evidence from China spanning 2001–2020

**DOI:** 10.1186/s12992-024-01025-z

**Published:** 2024-03-05

**Authors:** Afei Qin, Wenzhe Qin, Fangfang Hu, Meiqi Wang, Haifeng Yang, Lei Li, Chiqi Chen, Binghong Bao, Tianjiao Xin, Lingzhong Xu

**Affiliations:** 1 Centre for Health Management and Policy Research, School of Public Health, Cheeloo College of Medicine, Shandong University, Jinan, 250012 Shandong China; 2 National Health Commission (NHC) Key Laboratory of Health Economics and Policy Research (Shandong University), Jinan, 250012 Shandong China; 3 Center for Health Economics Experiment and Public Policy Research, Shandong University, Jinan, 250012 Shandong China

**Keywords:** Healthcare resources, Health inequality, Economic growth, Geographical distribution difference

## Abstract

**Background:**

There is a dearth of research combining geographical big data on medical resource allocation and growth with various statistical data. Given the recent achievements of China in economic development and healthcare, this study takes China as an example to investigate the dynamic geographical distribution patterns of medical resources, utilizing data on healthcare resources from 290 cities in China, as well as economic and population-related data. The study aims to examine the correlation between economic growth and spatial distribution of medical resources, with the ultimate goal of providing evidence for promoting global health equity.

**Methods:**

The data used in this study was sourced from the *China City Statistical Yearbook* from 2001 to 2020. Two indicators were employed to measure medical resources: the number of doctors per million population and the number of hospital and clinic beds per million population. We employed dynamic convergence model and fixed-effects model to examine the correlation between economic growth and the spatial distribution of medical resources. Ordinary least squares (OLS) were used to estimate the β values of the samples.

**Results:**

The average GDP for all city samples across all years was 36,019.31 ± 32,029.36, with an average of 2016.31 ± 1104.16 doctors per million people, and an average of 5986.2 ± 6801.67 hospital beds per million people. In the eastern cities, the average GDP for all city samples was 47,672.71 ± 37,850.77, with an average of 2264.58 ± 1288.89 doctors per million people, and an average of 3998.92 ± 1896.49 hospital beds per million people. Cities with initially low medical resources experienced faster growth (all β < 0, *P* < 0.001). The long-term convergence rate of the geographic distribution of medical resources in China was higher than the short-term convergence rate (|β_*i* + 1_| > |β_i_|, i = 1, 2, 3, …, 9, all β < 0, *P* < 0.001), and the convergence speed of doctor density exceeded that of bed density (bed: |β_i_| >doc: |β_i_|, i = 3, 4, 5, …, 10, *P* < 0.001). Economic growth significantly affected the convergence speed of medical resources, and this effect was nonlinear (doc: β_i_ < 0, i = 1, 2, 3, …, 9, *P* < 0.05; bed: β_i_ < 0, i = 1, 2, 3, …, 10, *P* < 0.01). The heterogeneity between provinces had a notable impact on the convergence of medical resources.

**Conclusions:**

The experiences of China have provided significant insights for nations worldwide. Governments and institutions in all countries worldwide, should actively undertake measures to actively reduce health inequalities. This includes enhancing healthcare standards in impoverished regions, addressing issues of unequal distribution, and emphasizing the examination of social determinants of health within the domain of public health research.

## Background

Since the millennium, there has been a significant improvement in global population health. However, since 2015, the pace of expanding access to basic healthcare services has slowed down, and inequalities still persist [1]. Firstly, there is a substantial disparity in healthcare resources (such as physician and hospital bed density) between countries. For instance, most low- and middle-income countries (LMICs) have not achieved universal coverage of basic healthcare throughout the life course [2]. Furthermore, there are considerable disparities in the distribution of healthcare resources within countries [3, 4]. These established facts raise a notable question: Does the geographical distribution of medical resources tend towards convergence or divergence during the process of economic growth?

Economic globalization has emerged as a significant trend in global economic development. This rapid development has encompassed various domains, including production, trade, finance, and investment, and has affected every aspect of the interconnected world economy. As one of the largest developing countries globally, China has experienced both opportunities and challenges as a result of economic globalization. Firstly, it contributes to attracting foreign investment. Secondly, economic globalization facilitates the acceleration of China’s industrialization process and the upgrading of its industrial structure. Thirdly, under the background of trade liberalization, it is conducive to a deeper engagement in international division of labor, leveraging the actual and potential comparative advantages of the country, and expanding overseas markets. Fourthly, economic globalization provides the conditions to seize the opportunities brought by the new technological revolution, harnessing China’s latecomer advantages, promoting the development of high-tech industries, and achieving rapid economic growth. In the context of economic globalization, overall, China’s economic development experience has set a good example globally. Since 2010, China’s Gross Domestic Product (GDP) has surpassed Japan, securing its position as the world’s second-largest economy. China’s per capita gross national product has also increased from around $300 in 1980 [5] to $12,741 in 2022 (based on annual average exchange rates) [6], a growth of over 42 times. However, the rapid economic growth has also led to an intensification of income inequality. China witnessed a significant rise in its Gini coefficient, soaring from 0.3043 in 1978 to a substantial 0.4624 in 2006 [7]. In 2010, China’s household Gini coefficient even reached a level of over 0.5, while the United States was at 0.45 [8]. It continued to exceed 0.460 from 2012 to 2015 [9]. Furthermore, the inequality gap in education, healthcare, and health has also widened [10]. In 2000, the World Health Organization (WHO) assessed and ranked the fairness of health financing and distribution among all member countries, with China ranking 188th out of 191 member countries [11]. Despite the rapid economic growth, there remains a discrepancy in the progress of population health and healthcare, which has not matched the same level of impressive advancements. According to some scholars, the rapid growth of China’s economy seems to have not yielded significant health benefits [12, 13]. Since the onset of economic reforms, the development of health in China has been less than satisfactory. Firstly, the improvement in life expectancy in underdeveloped provinces has lagged far behind those in wealthier provinces. Life expectancy is considered an important indicator of a nation’s public health status. According to population census estimates, from 1981 to 2000, the life expectancy in affluent cities such as Beijing and Shanghai increased by 4–5 years, rising from 71.9 years to 76.1 years and from 72.9 years to 78.1 years, respectively. In contrast, the improvement in life expectancy in Gansu, one of the poorest provinces in China, was much smaller, increasing by only 1.4 years during the same period, from 66.1 years to 67.5 years [14]. Therefore, prior to 2000, the disparity in health achievements among provinces with different levels of affluence undoubtedly widened. In addition, during the 1990s, the rate of increase in life expectancy in China was slower compared to other Asian countries with similar income and life expectancy levels, such as Indonesia and Malaysia [13]. Besides China, important gradients in health outcomes based on economic conditions have also been observed in other developing and developed countries, such as Spain [15], the United States [16], and Brazil [17]. In addition to intranational disparities, uneven economic development among nations also yields health inequalities associated with life expectancy. Findings from a study encompassing 115 countries indicate a clear association between income inequality and population health at the national level [18]. Furthermore, life expectancy levels in Africa and Southeast Asia are notably lower compared to the Americas, Europe, and the Western Pacific region [19]. Despite a sustained increasing trend in global life expectancy, evident spatial heterogeneity has emerged in the global distribution of life expectancy.

In addition to exacerbating inequalities in life expectancy, economic development appears to have brought about inequalities in other areas as well. Firstly, there is unfair access to clean energy. Two-thirds of the population in low- to middle-income countries live in regions where the Sustainable Development Goals for universal access to clean energy by 2030 are unattainable [20]. Secondly, there is unfair coverage of first-dose measles-containing vaccine (MCV1). In many low- to middle-income countries, progress in MCV1 coverage has stalled, with significant regional disparities, placing children at risk of preventable deaths. In some countries, coverage rates have even declined, exacerbating inequalities [21]. Thirdly, there are disparities in access to safe drinking water and sanitation facilities. Despite global improvements in access to safe drinking water and sanitation facilities from 2000 to 2017, disparities between low- to middle-income countries remain significant. In many countries, such improvements have coincided with worsening regional inequalities [22]. Fourthly, unfair occupational exposure. As many heavy industries and manufacturing sectors transition from high-income countries to LMICs, the adverse occupational exposures faced by high-income countries may manifest in future burdens in LMICs, such as asbestos exposure [23]. We do not know whether the exacerbation of inequality brought about by economic growth is temporary or permanent, and this is a matter worthy of deep consideration.

Furthermore, in the face of an economic crisis, there are also variations in healthcare expenditure across different countries. Research on the Group of Seven (G7) and Emerging Markets Seven (EM7) [24] reveals that during economic downturns, the growth rate of out-of-pocket medical expenditure in EM7 countries is not optimistic when compared to G7 countries, reflecting the vulnerability of impoverished regions. The actual GDP growth has a positive impact on out-of-pocket expenditures in G7 countries. However, in EM7 countries, it has a negative impact on the percentage of general government health expenditure as a share of current health expenditure (CHE), per capita CHE, and per capita out-of-pocket expenses. The actual GDP growth affects the healthcare expenditure patterns of both groups of countries and leads to diverging trends between them [24].

In addition, coming from the Global South, LMICs, especially the emerging market BRICS countries (Brazil, Russia, India, China, and South Africa), continue to be the engines of global real GDP growth [25]. They are all faced with the challenge of increasing burden of non-communicable diseases, accelerating population aging, and exceeding budgets for cutting-edge medical technologies [26]. Therefore, it is crucial to pay attention to the relationship between the economic development of these countries and the allocation of medical resources. Since the advent of economic globalization, the rapid economic development of the BRICS countries has gradually made them major world economies, and their share of healthcare expenditures has also been growing. However, they still face significant challenges in terms of health equity, non-communicable disease control, and improving population health [27], with serious ongoing inequalities. Taking China as an example, the allocation of public health resources across regions remains uneven. From 2008 to 2018, the ranking of public health resources in various provinces in China remained largely unchanged, with affluent provinces still ahead of underdeveloped provinces, and the imbalance in the distribution of public health resources between regions continues to widen [28]. Differences also exist within the BRICS countries, with Russia’s healthcare system being most exemplary, including improvements in national fertility rates, increased absolute healthcare expenditures, decreased mortality from chronic diseases, increased life expectancy, and reduced infant and maternal mortality rates [27]. Meanwhile, India is currently the fastest-growing major economy in the world, but according to the Gini coefficient in the report [29–31], there are significant inequalities within the country. In 1988, Brazil established the Sistema Único de Saúde with the aim of achieving universal health coverage and reducing disparities in the accessibility of health resources and health outcomes [32]. The establishment of SUS helped Brazil expand opportunities for all regions within the country to access healthcare services, reducing health inequalities [33]. However, there still exist multi-level geographic and socio-economic inequalities in health resources and outcomes [34]. Particularly since 2016, these disparities have further deteriorated [35].

The comprehensive improvement of a country’s healthcare capacity due to economic growth seems to be a consensus, but there are different opinions on whether the growth of medical resources is evenly distributed across all regions of the country. On the one hand, studies suggest that economic growth brings greater supply of doctors and hospital beds within a country, and the geographical distribution of healthcare resources tends towards convergence [36]. On the other hand, market failures manifest in the selection of work locations by doctors, as evidenced by their preference for cities with robust economic development [37]. These cities offer opportunities for doctors to capitalize on stronger market dynamics to stimulate patient demand, access superior social amenities, and benefit from an enhanced professional environment. These factors may hold greater influence in shaping location preferences than the potential market demand itself. Similarly, top-tier medical institutions are mostly located in large cities. Therefore, although medical resources increase, overall geographical inequality does not seem to decrease. Existing evidence also confirms this concern [4, 38–40]. In addition, The broadening of tax bases resulting from strong economic growth, flourishing domestic enterprises, and increased foreign direct investment in many emerging markets has not directly translated into increased health expenditure. In many regions, governments appear to have other priority areas, such as large-scale infrastructure projects (e.g., in Russia and Turkey), military expenditure (e.g., in Saudi Arabia), or other sectors such as tourism and agriculture (where healthcare costs are significant) [41]. Furthermore, it is worth noting that besides economic inequity, there may be other significant factors contributing to unequal distribution of healthcare resources. For instance, the scarcity of mental health resources in the Qinghai-Tibet Plateau region [42] and the difficulty in access to healthcare services for elderly Korean population due to limited transportation options [43]. In comparison to the northwest region of Sichuan Province in China, the southeastern region benefits from better transportation infrastructure, thus resulting in greater access to primary healthcare services [44]. Furthermore, residents living in more dispersed areas have fewer opportunities to access healthcare resources [45]. Factors such as natural geographical environment, transportation accessibility, population distribution, and other unexplored factors can also play important roles in the unequal distribution of healthcare resources. This study primarily focuses on the relationship between economic development and the allocation of healthcare resources, but it does not imply that other factors are less significant.

Fair allocation of medical resources is the foundation of establishing a robust healthcare system [46]. Government agencies and researchers worldwide have made long-term efforts to achieve healthcare equity. The WHO has emphasized the need for all countries to seek improvements in the fairness of healthcare service utilization, service quality, and financial protection for citizens during the exploration of universal health coverage [47]. However, previous studies on the spatial distribution of medical resources have mostly focused on provincial levels when studying at the national level [48], and non-national level studies often revolve around a single city [49] or region [50]. This is largely constrained by the limitations imposed by cities and regions. The same applies to studies on the accessibility of healthcare services, with the difference being the use of spatial location data for healthcare facilities. The existing studies based on provinces/states have laid the foundation for subsequent research combining geospatial big data with various statistical data [51, 52], but there is a lack of more detailed research based on samples from cities nationwide. Based on this, our study utilized medical resource and economic/population-related data for 290 cities in China to firstly investigate the dynamic geographic distribution patterns of healthcare resources in China, including physician density and hospital and clinic bed density. Additionally, we utilized a dynamic convergence model to examine the correlation between economic growth and the spatial distribution of medical resources. From the perspective of economic development, China’s economic growth not only has a global impact in terms of its speed and scale, but also in the unevenness of its development. There are significant disparities in development between the eastern and western regions of China, as well as between urban and rural areas. This imbalanced economic development pattern to a certain extent reflects the global economic disparities and inequalities among different regions worldwide. The unevenness of China’s economic development reflects the global issues of economic disparities between the North and the South, as well as between developed and developing countries. The findings of our study carry crucial implications for comprehending the inequalities in healthcare resource allocation within China and other developing nations, while also shedding light on the potential connections with economic development.

## Methods

### Data

The data used in this study were from the “China City Statistical Yearbook” for the years 2001 to 2020. The data in the yearbook was obtained from relevant departments of each city and the official annual data published by the National Bureau of Statistics (NBS) of China. The yearbook collected data for 300 cities in China, including direct-administered municipalities. We constructed a long panel dataset spanning from 2001 to 2020 based on these 300 cities. The inclusion criteria for urban samples were all cities accessible in the China Statistical Yearbook. The specific exclusion criteria for urban samples were as follows: 1) Cities not included in the China City Statistical Yearbook, including the Hong Kong Special Administrative Region, Macao Special Administrative Region, and Taiwan Province; 2) Cities with a lack of statistical data in numerous years, including Sansha, Lhasa, Kashgar, Qamdo, Nyingchi, Shannan, Nagqu, Hainan, Turpan, and Hami. Based on the above inclusion and exclusion criteria, this study included data from a total of 290 cities, including 4 direct-controlled municipalities (Beijing, Shanghai, Tianjin, and Chongqing), 15 vice-provincial cities (Guangzhou, Wuhan, Harbin, Shenyang, Chengdu, Nanjing, Xi’an, Changchun, Jinan, Hangzhou, Dalian, Qingdao, Shenzhen, Xiamen, and Ningbo), and 271 prefecture-level cities. According to the NBS’s provincial division criteria based on economic zones [53], the eastern region of China includes 10 provinces and municipalities (Beijing, Tianjin, Hebei, Shanghai, Jiangsu, Zhejiang, Fujian, Shandong, Guangdong, and Hainan); the central region includes 6 provinces (Shanxi, Anhui, Jiangxi, Henan, Hubei, and Hunan); the western region includes 12 provinces, municipalities, and regions (Inner Mongolia, Guangxi, Chongqing, Sichuan, Guizhou, Yunnan, Tibet, Shaanxi, Gansu, Qinghai, Ningxia, and Xinjiang); and the northeastern region includes Liaoning, Jilin, and Heilongjiang provinces. Due to the fewer number of provinces in the northeastern region, Liaoning was categorized in the eastern region, while Jilin and Heilongjiang were categorized in the central region. In summary, the 290 cities were divided into three regions based on their provinces, including 102, 101, and 87 cities in the eastern, central, and western regions, respectively. Additionally, for some cities, there were missing data for certain years due to administrative boundary adjustments and other reasons. Given the missing data for individual years in certain cities, we employed the Autoregressive Integrated Moving Average Model (ARIMA) for imputation. In conclusion, our study incorporated a sample size of 5763 observations, providing comprehensive data on healthcare resources, income levels, population size, and government expenditure across 290 cities.

In terms of outcome variables, healthcare resources were measured using two indicators: the number of physicians per million population and the number of hospital and clinic beds per million population. These indicators gauge the availability of healthcare professionals and the level of investment in healthcare infrastructure, which collectively reflect the overall extent of medical resources within cities. The explanatory variables consisted of nominal per capita GDP, population size, and local government expenditure from the general budget. These variables captured the impact of economic, population, and government-related factors on the expansion of healthcare resources.

We observed the changes in healthcare resources in China over a span of 20 years by describing the variations in the coefficient of variation (CV) of medical resources across cities. The CV quantifies the cross-sectional variability of the two outcome variables. It is computed using the following formula:

1 $${CV}_t=\sqrt{\frac{1}{N}\sum_{i=1}^N{\left(\frac{y_{it}-\overline{y_t}}{\overline{y_t}}\right)}^2},\overline{y_t}=\frac{1}{N}\sum_{i=1}^{i=N}{y}_{it}, for\ t=1,2,\dots, T$$

In Eq. (1), *y*_*it*_ represents the number of physicians and hospital beds per million people, while *N* denotes the total number of cities and *T* represents the years.

### The unconditional convergence model

In economic analysis, “convergence” refers to the tendency for income disparities between regions or countries to decrease over time. Convergence signifies the process of reducing differences in economic indicators as time progresses. Convergence includes σ-convergence and β-convergence, with β-convergence further categorized into unconditional β-convergence and conditional β-convergence. σ-convergence illustrates the trend of gradually diminishing differences in per capita income over time. β-convergence is utilized to analyze income mobility under similar conditions, where the growth rate of per capita income is negatively correlated with the initial level. Unconditional β-convergence does not include additional control variables, while conditional β-convergence does. In this study, β-convergence was employed to analyze the mobility of medical resource allocation under similar conditions, which meant that the growth rate of medical resources is negatively correlated with the initial level, thereby reducing the disparities in medical resource allocation between regions. We developed an unconditional convergence model to investigate the dynamic convergence of healthcare resources. The model is formulated as follows:

2 $$\mathit{\ln}\left(\frac{y_{i,\kern0.5em t+k}}{y_{i,t}}\right)=\alpha +\beta ln{y}_{i,t}+{\varepsilon}_{i,t}$$

In Eq. (2), y_i, t_ represents the total medical resources of city i in year t. α is the constant term and ε_i, t_ is the error term. The left side of the equation represents the growth rate of healthcare resources for the cities from year *t* to *t* + *k*, while the right side represents the initial stock of healthcare resources. This model only includes the initial stock of healthcare resources as explanatory variables, without any other conditional controls. The magnitude of the absolute value of *β* indicates the speed of convergence. A negative value of *β* suggests that cities with lower initial stock of healthcare resources experience faster growth. The parameter *k* represents the lagged years for measuring the growth rate.

### The conditional convergence model

Expanding upon the foundation of Eq. (2), we integrated the effects of economic, demographic, and government-related factors into the analysis of healthcare resource growth. Our main emphasis was on examining the impact of income growth (Eq. 3). To ensure the robustness of our findings, we also estimated a simplified model that solely considered per capita GDP-related variables (Eq. 4). Each model incorporated a set of dummy variables for different years to control for the effects of various factors such as inflation, policy changes, and so on. The specifications are presented as follows:

3 $$\mathit{\ln}\left(\frac{y_{i,t+k}}{y_{i,t}}\right)=\alpha +{\gamma}_0\mathit{\ln}{y}_{i,t}+{\gamma}_1\mathit{\ln}{Gdp}_{i,t}+{\gamma}_2\mathit{\ln}{Gdp}_{i,t}\bullet \mathit{\ln}{y}_{i,t}+{\gamma}_3{\left(\mathit{\ln}{Gdp}_{i,t}\right)}^2\bullet \mathit{\ln}{y}_{i,t}+{\gamma}_3\mathit{\ln}{Pop}_{i,t}+{\gamma}_4\mathit{\ln}{Gov}_{i,t}+{\delta}_t{\sum}_t^{t+k}{Year}_t+{\varepsilon}_{i,t}$$

4 $$\mathit{\ln}\left(\frac{y_{i,t+k}}{y_{i,t}}\right)=\alpha +{\beta}_0\mathit{\ln}{y}_{i,t}+{\beta}_1\mathit{\ln}{GDP}_{i,t}+{\beta}_2\mathit{\ln}{GDP}_{i,t}\bullet \mathit{\ln}{y}_{i,t}+{\delta}_t{\sum}_t^{t+k}{Year}_t+{\varepsilon}_{i,t}$$

In the Eqs. (3) and (4), city-specific variables are denoted as *Gdp*_*i*, *t*_, *Pop*_*i*, *t*_, and *Gov*_*i*, *t*_, signifying the per capita GDP, population (in 10,000), and local general budgetary expenditure of city i in year t, respectively. y_i, t_ represents the total medical resources of city i in year t. To mitigate the influence of extreme values, we apply natural logarithm transformations to these variables. Year_t_ represents year dummies. *γ*_0_ to γ_4_ and *β*_0_ to *β*_2_ represents coefficient of convergence. To explore the direct influence of per capita GDP on the convergence rate of healthcare resources in the model, we introduce interaction terms between per capita GDP and the initial level of healthcare resources (*γ*_2_ *ln Gdp*_*i*, *t*_ ∙  *ln y*_*i*, *t*_), as well as between the second order of per capita GDP and the initial level of healthcare resources (*γ*_3_(*lnGdp*_*i*, *t*_)^2^ ∙  *ln y*_*i*, *t*_). α is the constant term and ε_i, t_ is the error term. These additional terms allow us to investigate how per capita GDP impacts the convergence rate. A positive (negative) estimation of the coefficient *β*_2_ indicates that as income grows, there is a tendency for the convergence of healthcare resources to slow down (accelerate).

### Fixed effect model

In China, different provinces have distinct geographical, demographic, cultural, and economic characteristics. These features can impact both economic growth and the growth of healthcare resources. However, previous studies have rarely taken provincial-level heterogeneity into account, leading to biases in measuring and estimating convergence rates and economic impacts. In this study, we improved upon existing research by explicitly considering this unobserved heterogeneity among provinces using panel data. The following fixed effects models eliminated province-specific characteristics that remain constant over time:

5 $$\mathit{\ln}\left(\frac{y_{i,t+k}}{y_{i,t}}\right)=\alpha +{\gamma}_0\mathit{\ln}{y}_{i,t}+{\gamma}_1\mathit{\ln}{Gdp}_{i,t}+{\gamma}_2\mathit{\ln}{Gdp}_{i,t}\bullet \mathit{\ln}{y}_{i,t}+{\gamma}_3{\left(\mathit{\ln}{Gdp}_{i,t}\right)}^2\bullet \mathit{\ln}{y}_{i,t}+{\gamma}_3\mathit{\ln}{Pop}_{i,t}+{\gamma}_4\mathit{\ln}{Gov}_{i,t}+{\delta}_t{\sum}_t^{t+k}{Year}_t+{v}_i+{\varepsilon}_{i,t}$$

Compared to Eq. (3), the additional term *v*_*i*_ in Eq. (5) represents the provincial heterogeneity satisfying *E*[*v*_*i*_] = 0. By eliminating the unobserved provincial heterogeneity, the estimated coefficients of the model are more in line with the reality in China than those based on a cross-sectional data model.

### Data analysis

First, we described the mean, standard deviation, minimum, and maximum values of the total sample and three sub-samples (Eastern, Central, and Western regions). Subsequently, we proceed to compute the variations in the CV for healthcare resources across different cities. Finally, in the unconditional convergence model, we used Ordinary Least Squares (OLS) to estimate the values of β for the total sample and sub-samples. In the analysis, k ranged from 1 to 10. The analysis of sub-samples aimed to explore potential heterogeneity convergence patterns among high, medium, and low-income regions. The analytical approach for the conditional convergence model and the fixed effects model analysis methodology were the same as mentioned above. All data analyses were performed on Stata/MP version 17.0 and IBM Corp.’s SPSS software version 26.0. All *P* values were calculated using two-tailed tests, and those with a value of less than 0.05 were considered statistically significant.

Before conducting the analysis, we performed separate tests of stationarity and cointegration using the Levin-Lin-Chu and Kao tests for each model and variable. The test results indicated that the *P*-values for all model and variable tests were less than 0.001. Additionally, we conducted the Hausman test on the models, which resulted in rejecting the null hypothesis (*P* < 0.001), indicating that the fixed effects model was appropriate.

## Results

### The results of the descriptive analysis and the CV of healthcare resources across cities

The descriptive statistics results for the nationwide sample and three sub-samples were reported in Table 1. The nationwide sample consisted of 5739 observations. Among the 290 cities, 102 were classified as the Eastern region (2024 observations), 101 as the Central region (2010 observations), and 87 as the Western region (1705 observations). In the sample cities of 2001, the per capita GDP ranged from 1404 yuan to 152,099 yuan, with a mean and standard deviation of 9649.41 and 11,646.70, respectively. In the sample cities of 2020, the per capita GDP ranged from 17,430.00 yuan to 180,871.00 yuan, with a mean and standard deviation of 63,853.09 and 32,370.78, respectively. In the entire dataset, the per capita GDP in each city varied between 99 yuan and 467,749.00 yuan, with an average of 36,019.31 yuan. The number of doctors per million people and hospital beds per million people was 2016.31 and 3756.74 respectively. The average population size standed at 4.34 million, while the annual per capita fiscal expenditure amounted to 5986.28 yuan.

**Table 1:** Sample summary statistics of selected variables

Variable	2001				2020				Full sample			
	Mean	SD	Min	Max	Mean	SD	Min	Max	Mean	SD	Min	Max
Full Sample												
gdp	9649.41	11,646.70	1404.00	152,099.00	63,853.09	32,370.78	17,430.00	180,871.00	36,019.31	32,029.36	99.00	467,749.00
bed	2658.12	1234.18	570.44	8403.24	5155.46	1832.53	2246.58	13,518.83	3756.74	1783.33	570.44	13,765.88
doc	1650.36	849.72	538.66	7329.90	2878.30	1181.99	1111.26	8488.25	2016.31	1104.16	275.42	9833.17
gov	875.15	1364.37	220.42	19,697.06	12,854.09	7502.60	5272.36	67,635.61	5986.28	6801.67	220.42	109,377.74
pop	402.52	283.06	16.10	3097.91	460.83	332.28	21.11	3416.37	434.13	307.93	15.97	3416.37
obs	285				288				5739			
Eastern Region												
gdp	15,083.85	16,688.48	2647.00	152,099.00	79,029.51	38,307.63	27,577.00	165,851.00	47,672.71	37,850.77	2647.00	467,749.00
bed	2843.29	1380.38	881.85	8254.32	5318.71	1950.34	2246.58	12,208.70	3998.92	1896.49	813.75	13,765.88
doc	1652.01	826.72	570.24	6132.23	3314.24	1396.13	1335.83	8488.25	2264.58	1288.89	342.66	8845.09
gov	1303.63	2176.10	284.77	19,697.06	15,073.66	10,669.08	5272.36	67,635.61	7323.43	9258.02	284.77	109,377.74
pop	453.91	255.59	48.60	1327.14	529.31	303.47	64.58	1475.71	488.00	277.03	48.60	1475.71
obs	101				101				2024			
Central Region												
gdp	6813.66	4548.15	2269.00	42,886.00	55,800.29	22,957.68	22,122.00	131,441.00	29,377.73	23,249.08	2269.00	147,756.00
bed	2544.44	930.58	922.98	6735.91	4914.12	1545.65	2746.20	10,726.08	3543.83	1519.82	918.35	10,726.08
doc	1575.66	615.17	538.66	4151.79	2585.37	741.84	1532.28	6539.63	1868.19	820.66	375.54	7395.46
gov	539.24	207.40	220.42	1382.30	10,970.13	3467.46	5486.86	26,055.18	4749.28	4027.43	220.42	26,055.18
pop	404.26	229.67	69.70	1054.23	455.06	273.24	76.37	1256.69	434.78	254.96	69.70	1259.00
obs	101				100				2010			
Western Region												
gdp	6487.16	6966.15	1404.00	59,776.00	55,490.57	27,698.67	17,430.00	180,871.00	30,015.25	29,444.09	99.00	256,877.00
bed	2571.11	1352.71	570.44	8403.24	5243.35	1981.63	2395.11	13,518.83	3720.23	1895.10	570.44	13,518.83
doc	1739.24	1091.24	545.73	7329.90	2708.91	1187.61	1111.26	7486.87	1896.21	1107.06	275.42	9833.17
gov	762.51	491.77	239.73	3584.24	12,442.82	5618.18	5359.36	40,060.40	5857.24	5564.02	239.73	42,577.65
pop	337.87	355.00	16.10	3097.91	387.96	405.59	21.11	3416.37	369.43	379.36	15.97	3416.37
obs	83				87				1705			

The data is sourced from the “China Urban Statistical Yearbook 2001–2020”. The regional divisions of each province are determined by the State Council’s “Regional Development Plan”. *SD* standard deviation. *Min* minimum value. *Max* maximum value. The gdp is defined as GDP per capita (in Yuan), the bed is defined as number of hospital beds per million population, the doc is defined as number of physicians per million population, the gov is definded as annual government expenditures per capita, the pop is defined as population (in 10,000), and the obs is defined as sample size

Among the sub-samples, the Eastern cities exhibited the highest population size of 4.88 million, followed by the Central region with 4.34 million, and the Western region with 3.69 million. The per capita income in the Eastern region (46,672.71 yuan) was significantly higher than that in the Western region (30,015.25 yuan) and the Central region (29,377.73 yuan). Meanwhile, the Eastern region also had the highest number of doctors and hospital beds per million people (2264.58 and 3998.92, respectively), followed by the Western region (1896.21 and 3720.23) and the Central region (1868.19 and 3543.83). Lastly, a similar pattern was observed in the annual government expenditure of cities: the Eastern cities had the highest per capita expenditure (7323.43 yuan), followed by the Western cities with 5857.24 yuan, and the Central cities with the lowest public expenditure of 4749.28 yuan per capita. For detailed data on the years 2001 and 2020, please refer to Table 1.

**Fig. 1:**
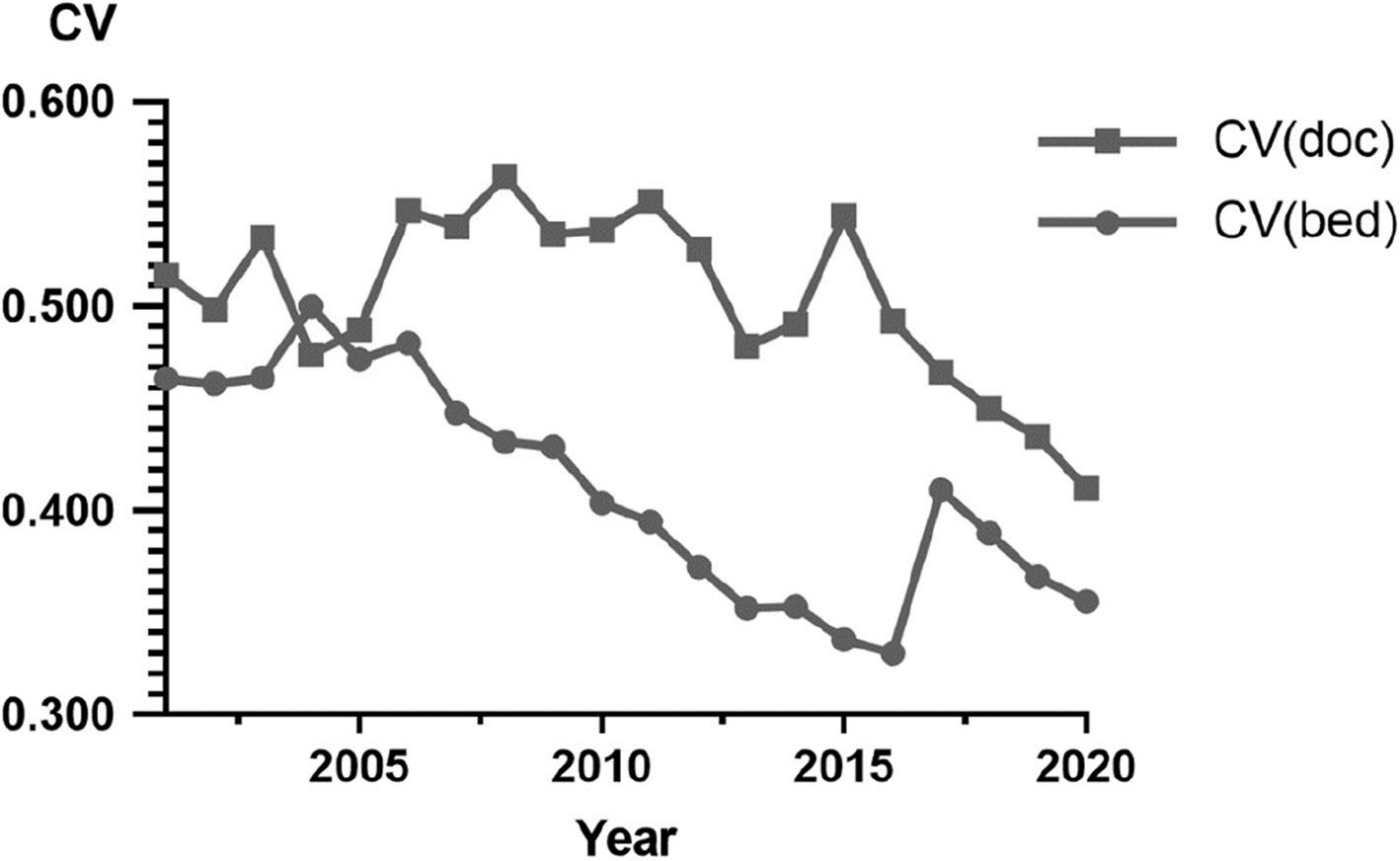
The variation in the CV for healthcare resources across cities from 2001 to 2020

Figure 1 illustrated the variation of the coefficient of variation (CV) for healthcare resources across cities. The decreasing CV for doctors and hospital beds indicated an overall trend of reduced cross-sectional dispersion of healthcare resources from 2001 to 2020, suggesting a more even distribution of doctors and hospital beds. It was worth noting that the CV for doctor density remained relatively stable around 0.5 from 2001 to 2015, followed by a sharp decline after 2015. The CV for hospital bed density exhibited an overall decline from 2001 to 2016, followed by a rise in 2017, and subsequent decreased.

### Unconditional convergence model results

The results of the unconditional convergence model were presented in Table 2. Firstly, for all 10 lag periods (*k* = 1 to *k* = 10), the estimated coefficients for *β* were both negative and statistically significant. This suggested a negative association between the growth rate of healthcare resources over *k* years and the initial level, which indicated that cities with initially limited healthcare resources underwent faster growth and gradually narrow the gap with cities that initially had abundant healthcare resources. Secondly, as the number of lag periods increased, the absolute values of the β coefficients also increased, suggesting a higher long-term convergence rate compared to the short-term rate. Thirdly, both doctor density and hospital bed density showed a similar convergence process with increasing absolute values of *β*. This implied a coordinated movement of human resources and capital in the distribution of healthcare resources. Lastly, regional differences in convergence rates existed. The Central region exhibited the highest convergence rate for doctor density, while the Western region demonstrated the highest convergence rate for hospital bed density. The patterns of change in doctor density and hospital bed density across the three regions with increasing lag periods were consistent with the overall sample.

**Table 2:** Regression results of unconditional convergence model based on the full sample and regional subsamples

Dependent Variable: ln (doc)		Lag1	Lag2	Lag3	Lag4	Lag5	Lag6	Lag7	Lag8	Lag9	Lag10
Full Sample	β	−0.173***	− 0.216***	− 0.255***	− 0.283***	− 0.316***	− 0.340***	− 0.367***	−0.392***	− 0.417***	− 0.434***
	SD	0.005	0.006	0.007	0.008	0.009	0.009	0.010	0.010	0.011	0.011
Western Region	β	−0.226***	− 0.257***	− 0.297***	−0.322***	− 0.357***	− 0.372***	− 0.396***	− 0.420***	− 0.433***	− 0.432***
	SD	0.011	0.013	0.015	0.017	0.018	0.019	0.020	0.021	0.022	0.022
Central Region	β	− 0.186***	− 0.265***	− 0.318***	− 0.364***	− 0.410***	−0.440***	− 0.469***	− 0.500***	− 0.528***	−0.559***
	SD	0.009	0.012	0.013	0.014	0.016	0.017	0.017	0.018	0.019	0.019
Eastern Region	β	−0.119***	−0.154***	− 0.187***	−0.212***	− 0.233***	−0.267***	− 0.302***	−0.329***	− 0.372***	−0.402***
	SD	0.007	0.008	0.010	0.011	0.012	0.013	0.014	0.015	0.015	0.016
R		0.171	0.216	0.255	0.283	0.316	0.340	0.367	0.392	0.417	0.434
R^2^		0.029	0.047	0.065	0.080	0.100	0.116	0.135	0.154	0.174	0.188
Adjusted R^2^		0.029	0.047	0.065	0.080	0.100	0.115	0.134	0.153	0.174	0.188
**Dependent Variable: ln (bed)**											
Full Sample	β	−0.142***	−0.202***	−0.268***	−0.334***	−0.375***	− 0.421***	−0.465***	− 0.508***	−0.542***	− 0.579***
	SD	0.003	0.004	0.005	0.006	0.006	0.007	0.007	0.008	0.008	0.008
Western Region	β	−0.156***	−0.213***	− 0.284***	−0.355***	− 0.403***	−0.459***	− 0.511***	−0.565***	− 0.599***	−0.639***
	SD	0.006	0.008	0.009	0.011	0.012	0.013	0.013	0.014	0.014	0.015
Central Region	β	−0.125***	−0.182***	− 0.243***	−0.305***	− 0.34***	−0.377***	− 0.420***	−0.461***	− 0.496***	−0.528***
	SD	0.005	0.007	0.009	0.01	0.012	0.013	0.014	0.015	0.016	0.016
Eastern Region	β	−0.147***	−0.211***	− 0.274***	−0.339***	− 0.377***	−0.416***	− 0.454***	−0.484***	− 0.519***	−0.558***
	SD	0.004	0.006	0.007	0.008	0.009	0.010	0.011	0.011	0.012	0.012
R		0.139	0.202	0.268	0.334	0.375	0.421	0.465	0.508	0.542	0.579
R^2^		0.019	0.041	0.072	0.111	0.141	0.177	0.217	0.258	0.294	0.335
Adjusted R^2^		0.019	0.041	0.071	0.111	0.140	0.177	0.216	0.258	0.293	0.335

***, **, and * indicate statistical significance at the 0.1, 1, and 5% levels, respectively

*SD* standard deviation

### Conditional convergence model results

Table 3 revealed that the estimated values of the initial stock of healthcare resources (*γ*_0_), while accounting for population, economy, government expenditure, and specific years, validated the prior findings and provided further evidence of the *β* convergence phenomenon in the distribution of healthcare resources in China. Nevertheless, it was observed that the marginal effect of estimating the initial stock of healthcare resources was relatively modest. This suggested a potential upward bias in the estimation of the convergence rate if other factors are disregarded.

**Table 3:** The results of the conditional convergence model regression

Dependent Variable: ln (doc)		Lag1	Lag2	Lag3	Lag4	Lag5	Lag6	Lag7	Lag8	Lag9	Lag10
year dummies	β	− 0.185***	− 0.232***	− 0.264***	− 0.298***	− 0.019	− 0.336***	− 0.323***	− 0.301***	− 0.273***	− 0.218***
	SD	< 0.001	< 0.001	< 0.001	< 0.001	0.001	< 0.001	< 0.001	< 0.001	< 0.001	< 0.001
ln (doc)	β	−1.461***	− 1.524***	− 1.488***	−1.499***	− 1.701***	−1.614***	− 1.724***	− 1.482***	− 1.390***	− 1.246***
	SD	0.101	0.119	0.130	0.140	0.152	0.157	0.164	0.168	0.175	0.181
ln (gdp)	β	−0.636*	− 0.481*	− 0.284	− 0.176	− 0.580*	− 0.212	−0.285	− 0.018	0.093	0.213
	SD	0.051	0.061	0.067	0.073	0.080	0.084	0.089	0.092	0.097	0.102
ln (gdp)*ln (doc)	β	2.637**	2.534**	2.279**	2.256**	2.724***	2.395**	2.646***	1.856*	1.517*	1.040
	SD	0.014	0.017	0.018	0.020	0.021	0.022	0.023	0.024	0.025	0.026
lngdp^2^*ln (doc)	β	−0.954*	−0.979*	−0.961*	−1.064**	− 0.896*	−1.122**	−1.236**	− 0.924*	− 0.791*	−0.553
	SD	< 0.001	0.001	0.001	0.001	0.001	0.001	0.001	0.001	0.001	0.001
ln (pop)	β	−0.018	−0.006	0.009	0.017	−0.022	0.027	0.035*	0.049**	0.057***	0.059***
	SD	0.004	0.004	0.005	0.005	0.006	0.006	0.006	0.006	0.007	0.007
ln (gov)	β	0.368***	0.435***	0.460***	0.500***	0.159***	0.521***	0.512***	0.499***	0.484***	0.438***
	SD	0.007	0.009	0.010	0.011	0.008	0.012	0.013	0.014	0.014	0.015
R		0.381	0.465	0.518	0.555	0.562	0.597	0.618	0.641	0.664	0.677
R^2^		0.145	0.217	0.269	0.308	0.316	0.356	0.382	0.411	0.441	0.458
Adjusted R^2^		0.144	0.216	0.268	0.307	0.314	0.355	0.380	0.410	0.440	0.457
**Dependent Variable: ln (bed)**											
year dummies	β	− 0.251***	− 0.320***	− 0.395***	− 0.435***	− 0.018	− 0.242***	− 0.143***	−0.065*	− 0.008	0.009
	SD	< 0.001	< 0.001	< 0.001	< 0.001	0.001	< 0.001	< 0.001	< 0.001	< 0.001	< 0.001
ln (bed)	β	−0.286	− 0.381	− 0.464	− 0.967**	− 1.040***	− 1.715***	−2.159***	−2.585***	−2.675***	−2.524***
	SD	0.064	0.083	0.100	0.116	0.131	0.140	0.148	0.153	0.158	0.163
ln (gdp)	β	0.930**	1.251***	1.352***	0.909**	0.474	−0.242	−1.034**	− 1.740***	−2.108***	− 2.196***
	SD	0.036	0.047	0.057	0.067	0.076	0.082	0.088	0.092	0.097	0.101
ln (gdp)*ln (bed)	β	0.203	0.497	0.691	2.211*	2.254*	4.225***	5.356***	6.405***	6.314***	5.412***
	SD	0.009	0.011	0.014	0.016	0.018	0.019	0.020	0.021	0.022	0.023
lngdp^2^*ln (bed)	β	−1.206**	−1.836***	−2.147***	−2.817***	−2.265***	−3.087***	−3.041***	−3.024***	− 2.498***	− 1.633***
	SD	< 0.001	< 0.001	< 0.001	< 0.001	< 0.001	0.001	0.001	0.001	0.001	0.001
ln (pop)	β	0.035*	0.054***	0.068***	0.074***	0.025	0.075***	0.076***	0.083***	0.095***	0.107***
	SD	0.002	0.003	0.003	0.004	0.004	0.005	0.005	0.005	0.005	0.006
ln (gov)	β	0.551***	0.685***	0.782***	0.772***	0.273***	0.558***	0.398***	0.262***	0.160***	0.077
	SD	0.004	0.006	0.007	0.008	0.006	0.01	0.01	0.011	0.012	0.012
R		0.314	0.401	0.455	0.493	0.475	0.513	0.529	0.555	0.580	0.607
R^2^		0.099	0.161	0.207	0.243	0.225	0.263	0.279	0.308	0.337	0.369
Adjusted R^2^		0.097	0.160	0.206	0.241	0.224	0.262	0.278	0.307	0.335	0.367

***, **, and * indicate statistical significance at the 0.1, 1, and 5% levels, respectively

*SD* standard deviation

In relation to the key explanatory variable, per capita GDP, the findings consistently demonstrated the estimated coefficients in the doctor density equation to be both negative and statistically significant across various lag indicators. This provided evidence of a pronounced trend towards increased doctor density in provinces with lower income levels. Moreover, our analysis revealed a positive and significant estimation for the parameter *γ*_2_, which represented the interaction between GDP and doctor density (except for *k* = 10). This indicated a direct opposing effect of the rising per capita GDP on the speed at which doctors converge. However, the estimated value of *γ*_3_, representing the interaction between the second-order measurement of GDP and the initial stock level, emerged as a negative and meaningful finding (excluding *k* = 10). This implied a nonlinear relationship in income convergence: as income grows, the initial effect is to impede the rate at which doctors converge, but a transition occurs where convergence begins to accelerate after surpassing a specific income threshold.

Regarding the equation for hospital beds, the estimated coefficients exhibited a similar pattern to the doctor equation in the long term (*k* > 3), but displayed differences in the short term (*k* ≤ 3). This observation suggested that *β* convergence in the distribution of hospital beds across cities took place over an extended timeframe, rather than in the immediate term. Furthermore, the estimate for the interaction term between the second-order GDP measure and the initial stock level consistently showed a negative and significant result across all lag periods. This underscored a nonlinear income convergence relationship: as income grows, the initial impact is to decelerate the rate of convergence for hospital beds, but a shift occurs after a certain income level is attained, leading to an accelerated convergence.

### Fixed effect model results

In the fixed effects model results presented in Table 4, most of the estimated coefficients exhibited similar signs as those shown in Table 3, with only a few variations in significance at individual lag periods. Additionally, the estimated values for the initial stock of doctors and hospital beds significantly decreased, indicating that the heterogeneity among provinces had a substantial impact on convergence. In the equation pertaining to doctors, the coefficient for the interaction term between per capita GDP and the initial stock exhibited a substantial decrease while maintaining its sign and significance with little alteration. The coefficients for the quadratic term of per capita GDP and the interaction term with the initial stock were not significant.

**Table 4:** The results of the fixed effects model regression

Dependent Variable: ln (doc)		Lag1	Lag2	Lag3	Lag4	Lag5	Lag6	Lag7	Lag8	Lag9	Lag10
year dummies	β	−0.004***	−0.004***	−0.004***	− 0.004***	−0.001	− 0.004***	− 0.005***	− 0.004***	−0.004***	− 0.003***
	SD	< 0.001	< 0.001	< 0.001	< 0.001	< 0.001	< 0.001	< 0.001	< 0.001	< 0.001	< 0.001
ln (doc)	β	−0.616***	−0.765***	− 0.808***	−0.894***	−1.003***	−1.045***	−1.130***	−1.011***	− 0.986***	−0.971***
	SD	0.103	0.126	0.138	0.148	0.162	0.167	0.164	0.177	0.182	0.179
ln (gdp)	β	−0.152**	−0.111	−0.053	− 0.038	−0.085	− 0.043	−0.073	0.027	0.06	0.048
	SD	0.052	0.067	0.074	0.080	0.089	0.093	0.090	0.101	0.106	0.103
ln (gdp)*ln (doc)	β	0.044**	0.044*	0.04*	0.044*	0.054*	0.051*	0.067**	0.043	0.039	0.042
	SD	0.014	0.017	0.019	0.020	0.022	0.023	0.023	0.024	0.025	0.026
lngdp^2^*ln (doc)	β	−0.001	−0.001	−0.001	−0.001	−0.001	−0.001	−0.002**	− 0.001	−0.001	− 0.001
	SD	< 0.001	0.001	0.001	0.001	0.001	0.001	0.001	0.001	0.001	0.001
ln (pop)	β	−0.003	0.003	0.009	0.013*	−0.011	0.022**	0.027***	0.035***	0.039***	0.036***
	SD	0.004	0.005	0.006	0.006	0.007	0.007	0.007	0.008	0.008	0.008
ln (gov)	β	0.071***	0.095***	0.118***	0.149***	0.042***	0.192***	0.219***	0.222***	0.232***	0.225***
	SD	0.008	0.011	0.012	0.013	0.009	0.015	0.015	0.017	0.017	0.016
AIC		− 3637.371	− 1975.381	− 1265.98	− 835.235	−337.375	− 423.285	−318.55	− 426.805	− 448.377	− 546.82
BIC		− 3617.57	− 1955.745	− 1246.518	− 815.958	−318.295	−404.415	−299.907	− 408.406	−430.243	−528.977
−2 log like lihood		− 3643.375	−1981.385	− 1271.985	− 841.24	− 343.381	− 429.291	− 324.556	−432.812	−454.385	−552.829
**Dependent Variable: ln (bed)**											
year dummies	β	−0.003***	−0.004***	−0.005***	−0.005***	−0.001	−0.003***	− 0.002***	−0.002***	− 0.001***	−0.001**
	SD	< 0.001	< 0.001	< 0.001	< 0.001	0.001	< 0.001	< 0.001	< 0.001	< 0.001	< 0.001
ln (bed)	β	−0.057	−0.093	0.184	0.051	−0.049	−0.431**	− 0.76***	−1.078***	−1.269***	−1.293***
	SD	0.065	0.084	0.104	0.120	0.136	0.143	0.151	0.155	0.158	0.162
ln (gdp)	β	0.134***	0.249***	0.560***	0.590***	0.543***	0.354***	0.119	−0.118	−0.323**	−0.440***
	SD	0.037	0.048	0.060	0.070	0.080	0.086	0.093	0.097	0.101	0.105
ln (gdp)*ln (bed)	β	−0.003	−0.005	−0.043**	− 0.028	−0.016	0.026	0.064**	0.102***	0.122***	0.118***
	SD	0.009	0.011	0.014	0.016	0.018	0.019	0.020	0.021	0.022	0.022
lngdp^2^*ln (bed)	β	−0.000*	−0.001**	−0.001*	−0.002***	− 0.002***	−0.003***	− 0.003***	−0.004***	− 0.004***	−0.003***
	SD	< 0.001	< 0.001	< 0.001	< 0.001	0.001	0.001	0.001	0.001	0.001	0.001
ln (pop)	β	0.008***	0.019***	0.033***	0.041***	0.013**	0.042***	0.040***	0.039***	0.040***	0.042***
	SD	0.002	0.003	0.004	0.005	0.005	0.006	0.006	0.006	0.006	0.006
ln (gov)	β	0.060***	0.104***	0.148***	0.170***	0.028***	0.149***	0.113***	0.079***	0.051***	0.028*
	SD	0.005	0.006	0.008	0.009	0.007	0.012	0.012	0.013	0.014	0.014
AIC		− 9835.489	− 6943.795	− 5241.642	− 4034.877	− 2839.135	− 2492.198	− 2151.791	−2030.466	− 1934.433	− 1898.389
BIC		− 9815.688	− 6924.159	− 5222.18	− 4015.599	− 2820.055	− 2473.328	− 2133.148	−2012.067	− 1916.299	− 1880.546
−2 log likelihood		− 9841.493	− 6949.800	− 5247.647	− 4040.882	− 2845.141	− 2498.204	− 2157.797	− 2036.473	− 1940.441	− 1904.397

***, **, and * indicate statistical significance at the 0.1, 1, and 5% levels, respectively. *SD*, standard deviation. The value of −0.000 ranges from −0.001 to −0.000007653. *AIC* denotes to Akaike information criterion. *BIC* denotes to Bayesian information criterion

In the equation concerning hospital beds, the coefficient for the interaction term between per capita GDP and the initial stock showed significance only for *k* ≥ 7, and exhibited a decline compared to the findings presented in Table 3. However, the coefficients for the quadratic term of per capita GDP and the interaction term with the initial stock remained significant and had the same signs as in Table 3, but with smaller absolute values. This suggested that the heterogeneity among provinces had a considerable impact on convergence during the process of economic growth.

## Discussion

This study was based on panel data from Chinese cities spanning from 2001 to 2020. By employing different models, we have demonstrated that the long-term convergence rate of the geographic distribution of healthcare resources in China was higher than the short-term convergence rate. Furthermore, the convergence rate of doctor density surpassed that of hospital bed density. Additionally, we have discovered that economic growth, measured by per capita GDP, significantly influenced the convergence rate of healthcare resources, and this effect was nonlinear. Lastly, the heterogeneity among provinces had an undeniable impact on the convergence of healthcare resources.

**Fig. 2:**
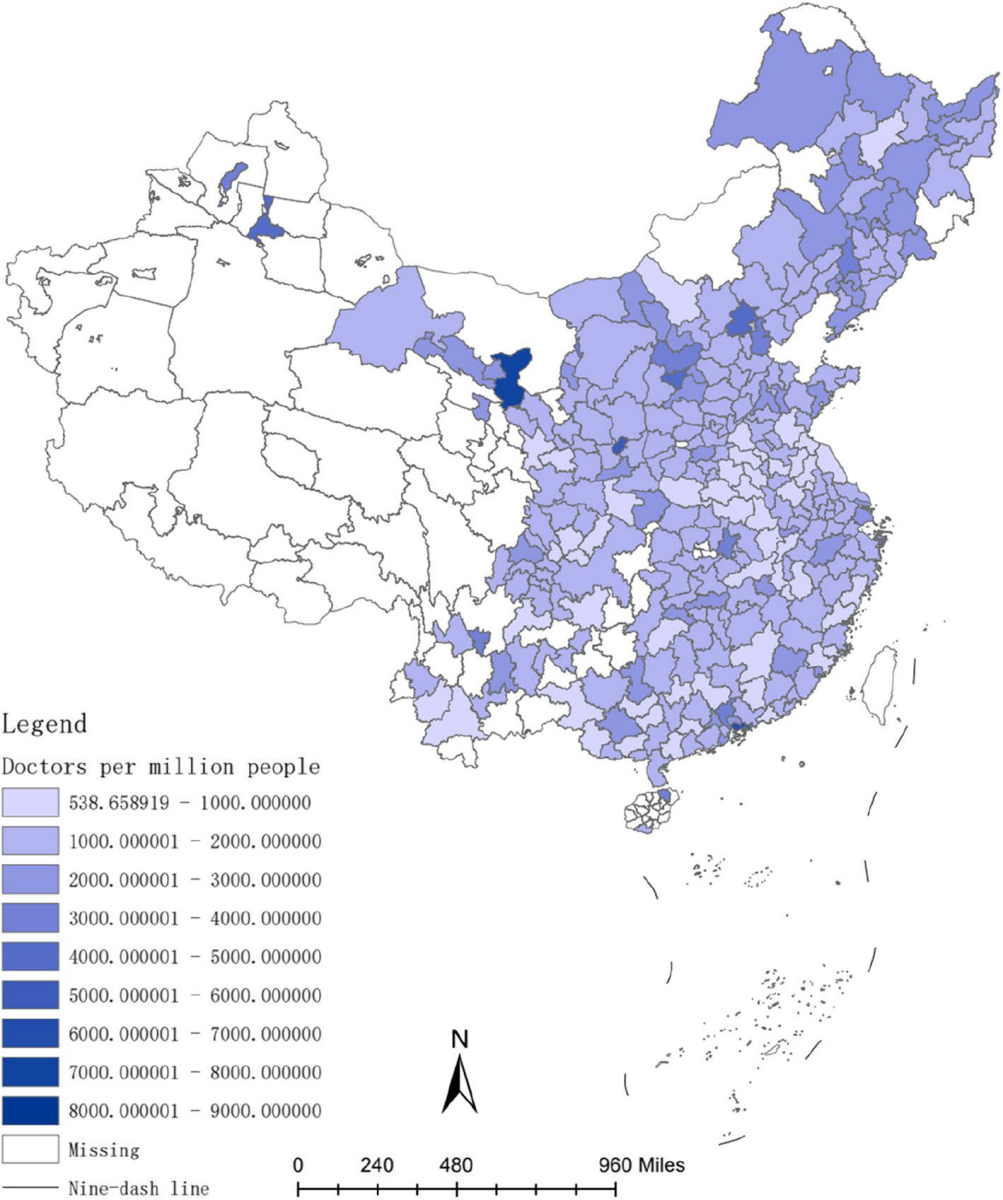
The physician density across Chinese cities in 2001

**Fig. 3:**
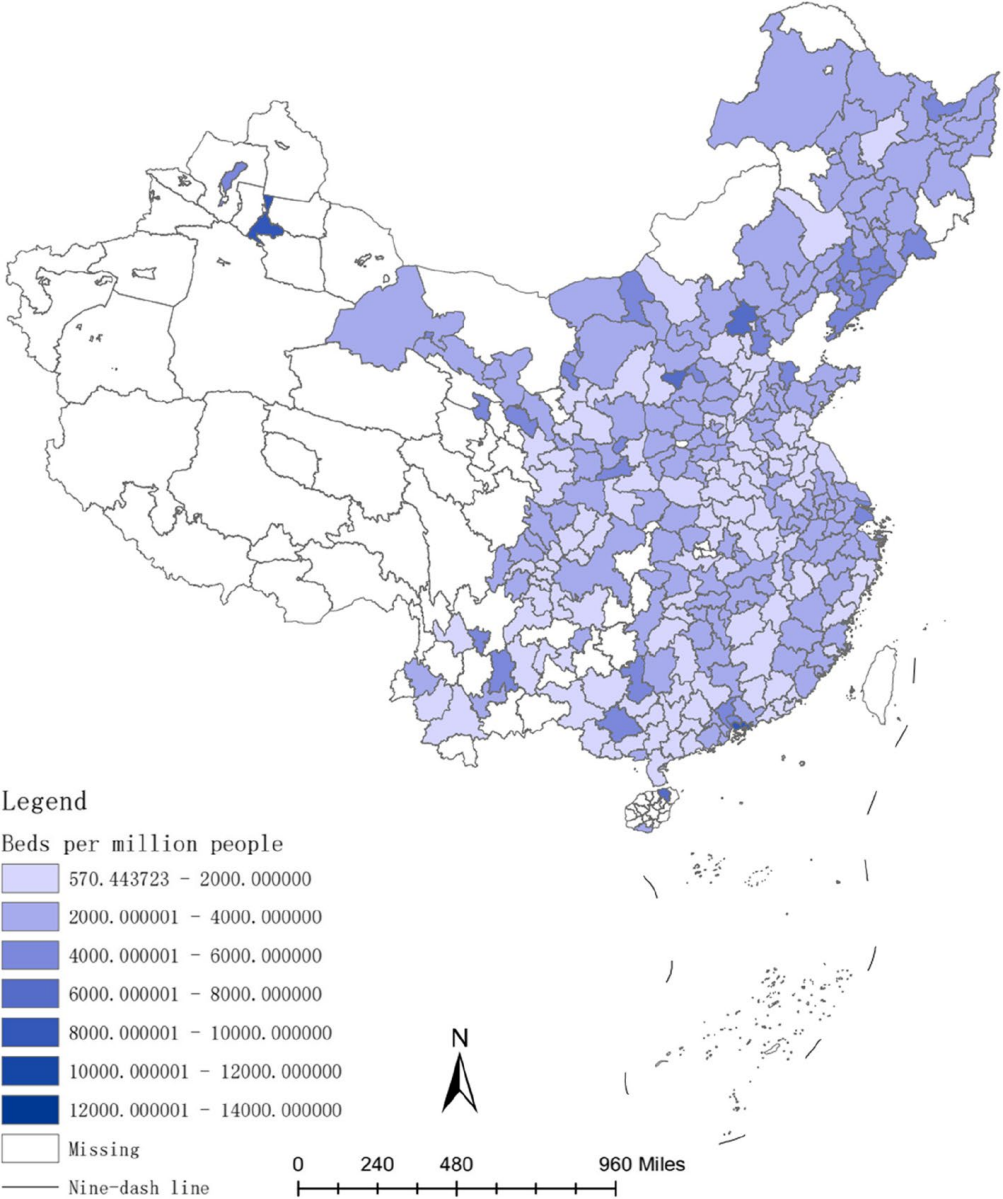
The hospital bed density across Chinese cities in 2001

**Fig. 4:**
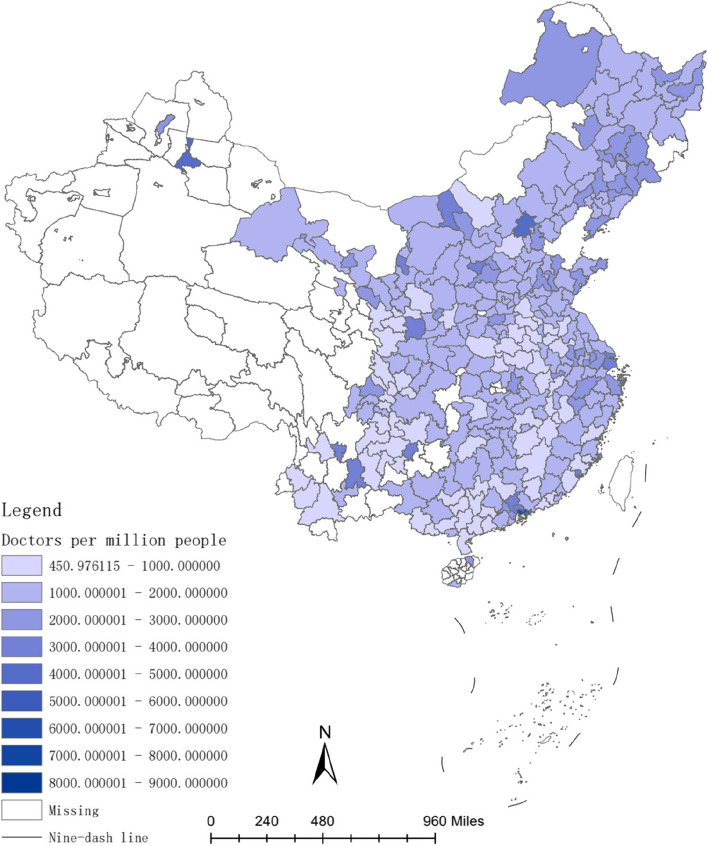
The physician density across Chinese cities in 2005

**Fig. 5:**
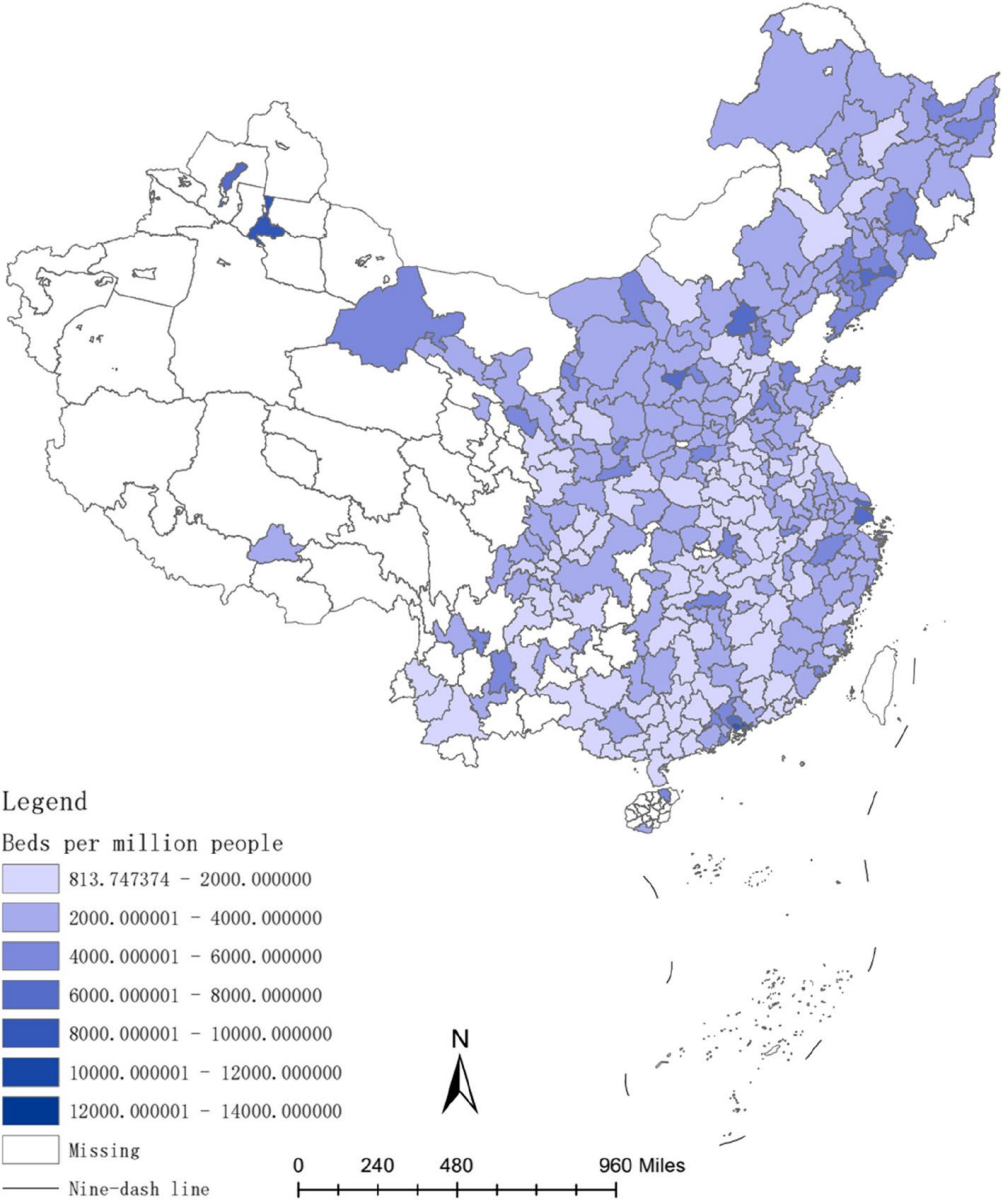
The hospital bed density across Chinese cities in 2005

**Fig. 6:**
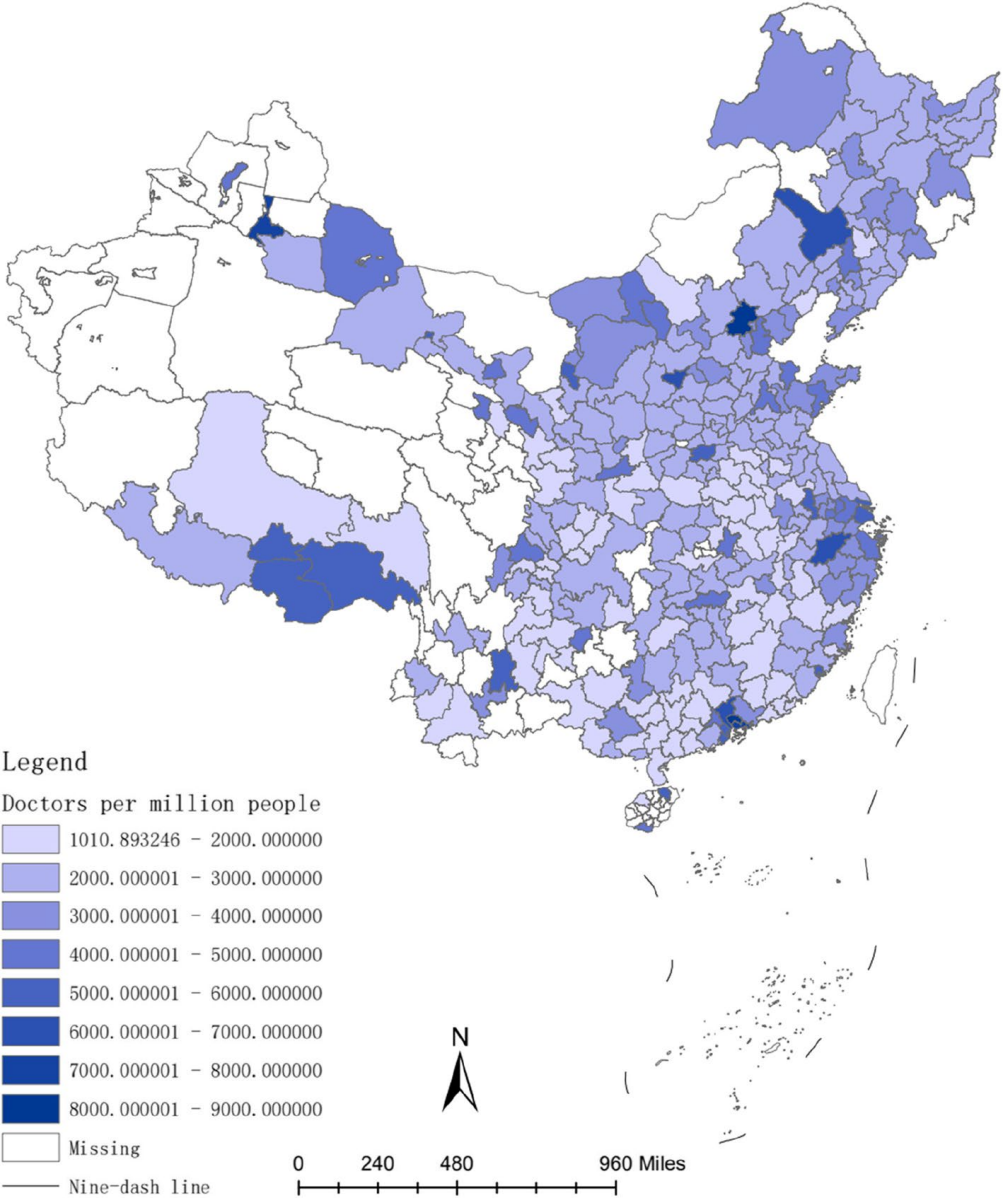
The physician density across Chinese cities in 2019

**Fig. 7:**
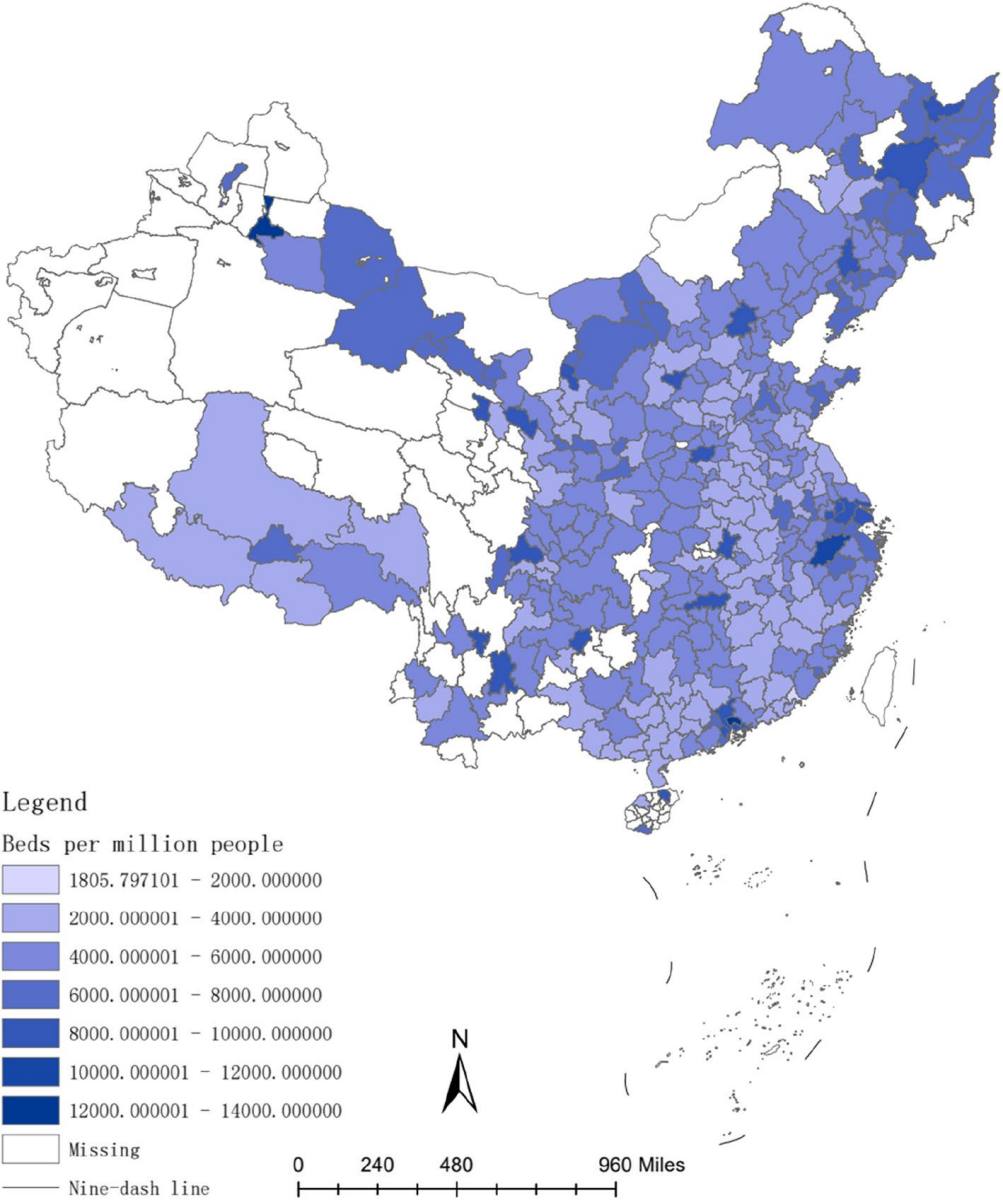
The hospital bed density across Chinese cities in 2019

The investment in healthcare resources, such as doctors and hospital bed, requires a lengthy process. For instance, doctors typically have higher educational levels compared to other professions [54], and their training period is often prolonged [55]. Therefore, the long-term convergence rate of investment in healthcare resources is higher than the short-term convergence rate. Figures 2, 3, 4, 5, 6 and 7 present the physician density and hospital bed density across Chinese cities in 2001, 2005, and 2019, respectively. From 2001 to 2005, there was no significant change in the distribution of healthcare resources among cities nationwide. However, from 2005 to 2019, there was a notable shift in the distribution of healthcare resources, indicating that the long-term convergence rate exceeds the short-term rate. Moreover, as time progressed, there was an increase in healthcare resource density, suggesting a positive correlation between per capita income and healthcare resource density. This finding aligns with the international trend where high-income countries generally possess more healthcare resources compared to low-income countries. Furthermore, between 2001 and 2019, there were dynamic changes in the distribution of healthcare resources, ultimately trending towards fairness.

The majority of doctors are usually employed in hospitals and primary healthcare institutions, with statistics showing that 92.3% of healthcare personnel in China work in hospitals and primary healthcare institutions [56]. Consequently, an increase in hospital beds leads to an increase in doctor density. In comparison to fixed capital investment, the adjustment speed of variable capital investment is faster and yields returns earlier. As hospital beds represent fixed capital, their convergence rate is lower than that of variable capital investment, i.e., doctors.

The influence of income growth on healthcare resources highlights the important role played by market mechanisms in guiding resource allocation. However, this process may not be linear. The findings provide evidence supporting the credibility of the Kuznets curve theory in elucidating the distribution patterns of healthcare resources in China. As per this theory, the occurrence of healthcare resource inequality among cities follows a pattern where it initially increases and subsequently decreases as per capita income experiences an upsurge. The Kuznets curve theory reflects the relationship between income inequality and economic development [57], and has subsequently been widely applied in other areas, such as healthcare and environmental quality [58, 59]. In the immediate term, economic growth in China engenders heightened disparities across various social dimensions, including healthcare. However, over the long haul, these disparities may diminish as healthcare resources converge among regions, consequently mitigating the unequal distribution of such resources. The inequality observed in the intercity allocation of healthcare resources manifests an inverted “U” trajectory, initially escalating in the short term before subsiding in the long term. It is worth noting that the outcomes presented in Table 3 indicate that sustained economic growth will reinforce the dynamic convergence pattern for the distribution of doctors. Nonetheless, given the sluggish adjustment pace of fixed capital, the distribution of hospital beds is still approaching a critical turning point, which will eventually reverse as per capita income continues to rise. In summary, our empirical research findings support the soundness of the Kuznets curve theory in expounding upon the healthcare resource distribution in China, wherein disparities initially augment across regions but subsequently decline as per capita income increases.

After incorporating the effects of provincial heterogeneity, the overall convergence rate is lower than the results without controlling for provincial heterogeneity. The convergence cycle of hospital bed density has been extended by 2 years (from *k* ≥ 4 to *k* ≥ 6). In comparison to per capita GDP, population size, and government expenditure, a substantial portion of the errors can be attributed to unobserved disparities in provincial characteristics. Nonetheless, these differences do not influence the overall rate of convergence. Decomposition of the estimates from the fixed model indicates substantial inter-provincial variations. Regional disparities in healthcare resources often result in patient mobility, which is a common phenomenon reflecting the status of healthcare reforms, legislative changes, and healthcare system development in a country or region [60]. Therefore, healthcare resources not only radiate within provinces but also have an impact on neighboring provinces. However, government regulations on the healthcare sector may hinder the transmission of market signals to the healthcare industry, thereby narrowing the supply-demand gap in healthcare resources and causing temporary surpluses or shortages of healthcare resources [61], which in turn reduces the cross-regional convergence of healthcare resources. Therefore, the empirical research outcomes we have obtained also indicate that a more lenient approach to policies within healthcare sector could potentially foster the seamless integration of healthcare resources throughout various regions, consequently aiding in the alleviation of healthcare disparities.

For a considerable period of time, economic development has exacerbated the inequitable distribution of healthcare resources. Without proactive measures, transitioning to the next stage may prove challenging. The poorest individuals among the poor suffer from a disproportionately high incidence of disease and premature death. A social gradient is observed across all countries: the lower the socioeconomic status, the poorer the health outcomes [62]. In other words, unequal economic status predicts unequal health outcomes. In the case of China, in addition to the widening disparities in health achievements among provinces with differing levels of prosperity as mentioned above, the differences in health outcomes resulting from wealth disparity are also pronounced. Although the under-5 mortality rate substantially decreased between 1996 and 2004, the rural areas still exhibited significantly higher rates than urban areas, with the mortality gap between rich and poor widening in rural areas. When categorizing rural areas according to their socioeconomic environments, the under-5 mortality rate in affluent rural areas decreased by approximately 50%, while the rate in relatively impoverished rural areas decreased by around 16% [63]. Before 2004, there was a significant disparity in the hospital delivery rates between urban and rural areas in China. However, the findings of this study demonstrate the credibility of the Kuznets curve theory in explaining the distribution patterns of healthcare resources in China. The economic development-induced wealth inequality is not a persistent trend; instead, it reaches a peak and subsequently begins to decrease. China has already experienced this turning point. The narrowing of economic inequality heralds the convergence of urban health resources, leading to a more equitable health outcome. For example, from 2000 to 2010, the life expectancy in Beijing and Shanghai increased by 4.08 and 2.12 years, respectively, from 76.10 and 78.14 years to 80.18 and 80.26 years. In Gansu Province, life expectancy increased by 4.76 years, from 67.47 to 72.23 years, surpassing the growth rate of the two wealthiest regions in China [64]. Furthermore, following China’s proposal for deepening healthcare reform in 2009, urban healthcare resources have been gradually converging, resulting in a diminishing urban-rural divide and a reduction in health outcome disparities attributable to economic disparities [12]. For instance, after 2009, the in-hospital delivery rates in both urban and rural China remained at relatively high levels with little differentiation, reaching 99.9% in 2019 [64]. These data indicate that, as the economic level advances, disparities in healthcare resources between urban areas initially increase before subsequently decreasing.

Governments and institutions in China, as well as across all nations globally, should actively take steps to address health inequities. Indeed, China is making efforts in this regard. In 2013, China launched the targeted poverty alleviation program with the goal of eradicating extreme poverty by 2020. In 2016, China issued *Healthy China 2030* plan, aiming to achieve high-quality universal health coverage by 2030. In 2019, *Healthy China Action (2019–2030)* was released. These robust economic and health policies have yielded results in the healthcare sector, including the promotion of inter-regional medical insurance policies and strong support for the construction of national regional medical centers [65, 66]. For instance, as of July 2023, China has identified 125 projects for the construction of national regional medical centers in five batches, covering provinces with weak healthcare resources [67]. We are confident that in the future, the unequal distribution of healthcare resources in China will gradually decrease. The Russian government has also taken proactive steps and made efforts. Although Russia experienced a severe economic downturn after the dissolution of the Soviet Union in 1991 [68], and had very low healthcare expenditure, improvement began rapidly in 2000. The government increased spending on healthcare and on vulnerable populations, alleviating the vulnerability of rural and low-income groups to catastrophic healthcare expenses [69]. Moreover, early insurance coverage heavily depended on geographical location and employment status, with higher coverage among the affluent population. However, this trend gradually evolved into a more equal distribution, with almost 97.4% of the Russian population having medical insurance by 2011 [70]. India lags behind most neighboring countries in life expectancy, maternal mortality rate, and infant mortality rate, and many people struggle to access adequate and affordable care [71]. In light of this disadvantage, India has also been actively exploring new healthcare models to promote health equity. In 2007, India launched the Rashtriya Swasthya Bima Yojana (RSBY), a program that provides free medical services to impoverished families, although the program has faced many shortcomings. In 2010, India proposed detailed reform plans to expand RSBY. In 2017, based on RSBY, India announced a healthcare program called “Ayushman Bharath,” consisting of two parts: strengthening existing primary healthcare and providing inpatient treatment for identified low-income families. India has committed to achieving universal health coverage, although progress has not been effectively improved at present and faces significant challenges [71]. The health disparities between countries have also been alleviated, in addition to within each country. Some forecasts indicate that the competitiveness of the BRICS countries (Brazil, Russia, India, China) has been continuously strengthened compared to the major markets of the Organization for Economic Cooperation and Development (OECD) [72]. From 1995 to 2012, the share of global health expenditure accounted for by the OECD declined significantly in nominal and purchasing power parity terms [72], indicating a certain improvement in the unfair distribution of health resources between high-income and middle-to-low-income countries.

Therefore, we advocate that all countries around the world should actively implement corresponding measures, which includes improving the birth environment and healthcare standards in impoverished areas and among vulnerable populations, addressing inequalities in rights, finances, and resource allocation, establishing a system for monitoring health equity, conducting internal assessments of health inequality, investing in training for decision-makers and healthcare professionals, and emphasizing social determinants of health in public health research. Despite facing numerous challenges, China’s experience provides important insights. Since 2013, the Chinese government has proposed and implemented the “Belt and Road” cooperation initiative, aiming to connect the Asia-Pacific economic area with the European economic area through the establishment of the “Silk Road Economic Belt” and the “21st Century Maritime Silk Road,” thereby creating a significant practice of building a community with a shared future for mankind. This initiative provides a platform for strengthening global health strategies.

Addressing the inequitable distribution of healthcare resources between different cities is a complex issue that requires collaborative efforts from the government, healthcare institutions, and society as a whole. In response to this, we propose several possible approaches: 1) Establishing equitable resource allocation standards: Develop scientifically-based healthcare resource allocation standards that take into account factors such as population density, regional development level, and disease burden, ensuring fair distribution of healthcare resources. 2) Increasing investment in primary healthcare services: Enhance investment in primary healthcare facilities in rural and remote areas, improving their service capabilities to bridge the healthcare resource gap between urban and rural areas. 3) Improving the mobility of healthcare services: Encourage healthcare professionals, including doctors and nurses, to move between different regions, particularly to disadvantaged and densely populated areas, to balance the supply of medical resources across different regions. 4) Enhancing healthcare insurance policies: Strengthen healthcare insurance coverage for low-income groups, alleviating the burden of medical expenses and enhancing their access to healthcare services. 5) Strengthening regulation and assessment: Establish sound regulatory systems to supervise and evaluate the allocation of healthcare resources, promptly identifying and rectifying any unfair practices. 6) Enhancing transparency in healthcare resource information: Establish a comprehensive system for publicly disclosing healthcare resource information, including the actual situation of healthcare resources, distribution criteria, and utilization, ensuring that the public and regulatory authorities have a clear understanding of the allocation of healthcare resources. 7) Promoting integrated utilization of healthcare resources: Facilitate the integration and utilization of healthcare resources between different cities, by establishing platforms for sharing healthcare resources, ensuring their more efficient and effective utilization. 8) Encouraging tilt of medical technology and resources towards underdeveloped areas: The government can incentivize the tilt of medical technology and resources towards underdeveloped areas through policies such as establishing dedicated medical aid funds to support the healthcare resource development and technological upgrades in these regions. Furthermore, as described in the background, apart from uneven economic development, other factors such as natural geographic environment, transportation convenience, and population distribution may also be important reasons affecting the distribution of healthcare resources. Therefore, it is necessary to work together from various perspectives and approaches to promote fair distribution of healthcare resources. In summary, improving the inequitable distribution of healthcare resources between different cities requires comprehensive strategies, including the formulation of reasonable policies, strengthened regulation, promotion of resource sharing and integrated utilization, to achieve equitable distribution and maximized utilization of healthcare resources.

Our study has several limitations. Firstly, we focused only on the density of healthcare resources, while the distribution of healthcare resource quality is equally important to investigate. Secondly, the underlying mechanisms through which GDP influences healthcare resources remain unclear, such as the preference of physicians and hospitals for location selection. Thirdly, our study employed physician density and bed density as proxies for healthcare resources, which cannot differentiate whether the allocation of specialized physicians and other resources is fair. In fact, the distribution of different specialized physician groups can vary significantly. Future research can explore the quality of healthcare resources and examine different specialized physicians, which may have important policy implications for ensuring equitable access to high-quality healthcare services for both urban and rural populations.

## Conclusions

Our research findings present three important policy implications. Firstly, cities with initially underdeveloped healthcare resources experience faster growth and gradually catch up with cities that initially had abundant healthcare resources. Secondly, there exists a convergence relationship between income growth and healthcare resources among cities, and this relationship is nonlinear. Although temporary effects may generate heightened inequality, the process of healthcare resource convergence represents a long-term phenomenon, necessitating a considerable time investment to address and rectify inequality. Lastly, the geographical dependence across provincial boundaries may alleviate the healthcare resource inequality resulting from income disparities. Governments and institutions in China, as well as in all countries worldwide, should actively undertake measures to actively reduce health inequalities. This includes enhancing healthcare standards in impoverished regions, addressing issues of unequal distribution, and emphasizing the examination of social determinants of health within the domain of public health research.

## Data Availability

The datasets generated and analysed during the current study are not publicly available due internal sharing regulations of Shandong University but are available from the corresponding author on reasonable request. The data used in this study are also publicly available through national statistical offices, national or regional statistical yearbooks and specific literature.

## Abbreviations

LMICs: low- and middle-income countries
GDP: Gross Domestic Product
WHO: World Health Organization
MCV1: first-dose measles-containing vaccine
G7: Group of Seven
EM7: Emerging Markets Seven
CHE: current health expenditure
NBS: National Bureau of Statistics
ARIMA: Autoregressive Integrated Moving Average Model
CV: Coefficient of Variation
OLS: Ordinary Least Squares
RSBY: Rashtriya Swasthya Bima Yojana
OECD: Organization for Economic Cooperation and Development
